# New Perspectives on Antimicrobial Agents: Sulbactam-durlobactam

**DOI:** 10.1128/aac.01086-24

**Published:** 2025-08-22

**Authors:** Jason M. Pogue, Jeffrey Harrington, Walaiporn Wangchinda, Keith S. Kaye

**Affiliations:** 1 Department of Clinical Pharmacy, University of Michigan College of Pharmacy 15514, Ann Arbor, Michigan, USA; 2 Department of Pharmacy, University of Michigan Health 21707, Ann Arbor, Michigan, USA; 3 Department of Medicine Siriraj Hospital, Mahidol University 26685, Bangkok, Thailand; 4 Division of Allergy, Immunology and Infectious Diseases, Rutgers Robert Wood Johnson Medical School 12287 , New Brunswick, New Jersey, USA; Samsung Medical Center, Seoul, Republic of Korea

**Keywords:** sulbactam, durlobactam, CRAB, *Acinetobacter baumannii*

## Abstract

Infections due to Carbapenem-resistant *Acinetobacter baumannii* (CRAB) are a significant burden and are associated with devastating outcomes. Subsequently, CRAB is recognized as an urgent threat to human health by the United States (US) Centers for Disease Control and Prevention and is designated a critical pathogen, with the highest priority for research and development of novel treatment options, by the World Health Organization. Sulbactam-durlobactam was approved by the US Food and Drug Administration in 2023 for the treatment of Hospital Acquired and Ventilator Associated Bacterial Pneumonia caused by susceptible strains of *A. baumannii*, and as of 2024, is the Infectious Diseases Society of America Guidance on the Treatment of Antimicrobial Resistant Gram-Negative Infections preferred treatment strategy, in combination with a carbapenem, for infections due to CRAB. In this commentary, we will dive into the preclinical and clinical evidence supporting this approval. We will further explore controversial and emerging questions related to sulbactam-durlobactam, including the necessity of carbapenem combination therapy for CRAB and the other potential therapeutic opportunities durlobactam may provide beyond *A. baumannii*.

## PERSPECTIVE

*Acinetobacter baumannii* is a challenging nosocomial pathogen due to its propensity for causing severe infections in hospitalized patients and its wide array of resistance mechanisms to the most commonly used antibiotics. *A. baumannii* is predominantly associated with infections involving the respiratory tract (hospital-acquired bacterial pneumonia [HABP] and ventilator-associated bacterial pneumonia [VABP]), chronic and injury-related wounds, and in the bloodstream of patients with central venous catheters (1). In-hospital mortality rates for hospitalized patients with infections due to *A. baumannii* have been estimated at 13.6%, but a higher mortality rate of 33.6% has been observed in ICU patients with bloodstream infections (2, 3). Unsurprisingly, clinical outcomes are significantly worse among patients with infections due to carbapenem-resistant *A. baumannii* (CRAB) compared to susceptible infections, given both delays in starting appropriate antibiotics and the limited treatment options available (4). This is exacerbated by the ever-increasing prevalence of carbapenem resistance amongst *A. baumannii*, which rose from 11.2% to 40.3% in North America from 1997 to 2016 (5), and has eclipsed 80% in parts of Latin America, Eastern Europe, and the Asia-Pacific region as of 2019 (6). In light of this, the United States Centers for Disease Control and Prevention has recognized CRAB as an urgent threat to public health (7), and the World Health Organization has designated CRAB a critical pathogen, with the highest priority for research and development of novel treatment options (8).

Sulbactam-durlobactam has been approved for use in the United States by the FDA in 2023 for HABP and VABP caused by susceptible strains of *A. baumannii*. The scope of this commentary is to provide an overview of the role of sulbactam-durlobactam for the treatment of infections due to CRAB. We will also explore controversial and emerging questions related to sulbactam-durlobactam, including the necessity of carbapenem combination therapy for CRAB infections, as well as the other potential therapeutic opportunities durlobactam may provide beyond *A. baumannii*.

## THE IDSA GUIDANCE DOCUMENT APPEARS QUITE BULLISH ON HIGH DOSE SULBACTAM, DO I EVEN NEED AN INHIBITOR?

Sulbactam is a penicillanic acid sulfone beta-lactamase inhibitor (BLI) with relatively limited irreversible inhibition of Ambler Class A and C beta-lactamase enzymes. With respect to *A. baumannii*, sulbactam displays intrinsic antibacterial activity. Sulbactam exerts its antibacterial activity against *A. baumannii* through direct inhibition of penicillin-binding protein 3 (PBP3), and to a lesser extent, PBP1a/1b (9). With limited treatment options available for severe CRAB infections, high-dose ampicillin-sulbactam has been one of the mainstays of combination therapy and is recommended as a therapeutic strategy in clinical guidelines (10–12).

Importantly, however, carbapenem resistance is frequently associated with reduced *in vitro* susceptibility rates for sulbactam (13, 14). As susceptibility for sulbactam alone is only tested in combination with its inactive partner, ampicillin, it is important for clinicians to understand that it is the denominator of the ampicillin-sulbactam minimum inhibitory concentration (MIC) (e.g., 2 when the MIC is 4/2 mg/L) that represents the sulbactam MIC. Among 10,749 *A*. *baumannii* isolates tested for ampicillin-sulbactam susceptibility in the SENTRY program, 37.4% were susceptible, applying the CLSI breakpoint of MIC ≤8/4 mg/L (i.e., 4 mg/L of sulbactam) (13). However, in the subset of 6,541 isolates that tested imipenem-non-susceptible, the cumulative susceptibility of sulbactam decreased dramatically to 4.5%. These findings are consistent with the poor sulbactam susceptibility rates demonstrated in CRAB isolates from patients with ICU-acquired pneumonia. In the ATLAS database consisting of 2,905 global meropenem-resistant (MIC > 8 mg/L) respiratory *A. baumannii* isolates collected from ICU patients between 2014 and 2021, only 5.7% of isolates tested susceptible to sulbactam, with reported MIC_50_ and MIC_90_ values of 32 and ≥64 mg/L, respectively (14). Taken together, these findings suggest that the mechanisms underlying reduced carbapenem susceptibility are also responsible for the loss of *in vitro* potency of sulbactam. In particular, the subgroups of Class D carbapenem-hydrolyzing OXA enzymes that are prevalent in CRAB, most notably the OXA-23 subgroup, as well as the respective Class A and C TEM-1 and ADC enzyme groups, are capable of efficiently inactivating sulbactam and are likely driving the elevations in MICs demonstrated.

The question is whether standard or aggressive dosing of ampicillin-sulbactam can overcome this elevated MIC distribution in CRAB. Recently ascertained PK/PD data help answer this question. The PK/PD profile of sulbactam in *A. baumannii* has been determined in both *in vitro* and *in vivo* (animal) models. Yokoyama et al. utilized a sulbactam-susceptible (sulbactam MIC 0.5 mg/L) strain to identify the PK/PD index best correlated with bacterial killing and the exposures necessary to achieve antibacterial activity (15). In both the thigh and lung models, fT > MIC was most strongly correlated with the efficacy of sulbactam. Further assessment of the magnitude of fT > MIC of sulbactam required for bacterial stasis and 1-log_10_ kill demonstrated that target values of 21.0% and 32.9% in the thigh model and 20.4% and 24.5% in the lung model, respectively, were associated with these levels of activity. While informative, this assessment was limited by the use of a single, susceptible reference strain. More recently, sulbactam PK/PD in *A. baumannii* has been rigorously evaluated in a murine lung model incorporating data from 21 clinical isolates with sulbactam and meropenem MICs ranging from 1 to 32 mg/L and 0.25 to >64 mg/L, respectively (16). While fT > MIC was again identified as the PK/PD index most strongly correlated with antibacterial activity, a distinct, bimodal distribution of fT > MIC magnitude associated with antibacterial activity was identified between the 17 isolates that were reported as susceptible or intermediate to either sulbactam or meropenem, and the four isolates that were reported resistant to both agents. Among the 17 isolates that were susceptible or intermediate to at least one agent, median fT > MIC values of 11.6% and 21.2% were necessary to achieve stasis and 1-log_10_ kill with sulbactam, respectively. However, the four carbapenem-resistant isolates with elevated sulbactam MICs (>8 mg/L) required considerably higher sulbactam fT > MIC exposures of 50.6% and 60.4% to achieve the same effect. A Monte Carlo simulation (MCS) was then performed using these targets with population PK parameters of sulbactam from 16 critically ill patients with *A. baumannii* VABP (17). In this MCS, a lower target of 21.2% fT > MIC was applied for isolates susceptible or intermediate to sulbactam, whereas the higher target of 60.4% was used for sulbactam-resistant isolates (16). The analysis demonstrated that both standard-dose (1 g q6h infused over 0.5 h) and high-dose (3 g q8h infused over 4 h) sulbactam can achieve high probabilities of target attainment (PTA) of ~90% or higher up to an MIC of 8 mg/L (sulbactam-intermediate). However, neither regimen could achieve PTA above 60% in sulbactam-resistant isolates owing to the substantial increase in the fT > MIC target when moving to this category. Therefore, while the Infectious Diseases Society of America (IDSA) has suggested high-dose sulbactam-based therapy as part of combination regimens regardless of sulbactam MIC, it is important to note that this was only the *preferred* strategy for CRAB management prior to the availability of sulbactam-durlobactam, and these recent PK/PD findings, in combination with the relative absence of clinical data supporting sulbactam in the setting of sulbactam resistance, highlight the critical need for better options.

## SULBACTAM-DURLOBACTAM TO THE RESCUE?

Durlobactam is a novel diazabicyclooctanone (DBO) BLI that was designed through modifications to the bicyclic core structure of avibactam, with the goals of improving inhibitory activity against Class A and C enzymes, extending the spectrum of inhibition to include a wider range of Class D enzymes, and enhancing Gram-negative cell permeation (18). Durlobactam, when compared to avibactam, demonstrates 10-fold IC_50_ (half maximal inhibitory concentration) reductions against both KPC-2 and AmpC and nearly a 100-fold IC_50_ reduction against OXA-24. The translation of potent Class D activity to *in vitro* bacterial growth inhibition is supported by >4-fold MIC reductions observed in isogenic strains of *A. baumannii* overexpressing OXA-23 and OXA-24 when durlobactam was added independently to piperacillin, meropenem, and sulbactam at a fixed concentration of 4 mg/L (18).

Although durlobactam is capable of selectively inhibiting PBP2 in *A. baumannii,* leading to alterations in cell morphology, it lacks independent antibacterial activity against this organism with an MIC_50_ of >64 mg/L (18, 19). However, durlobactam potently restores the activity of sulbactam through beta-lactamase inhibition, lowering the sulbactam MIC_90_ 32-fold, from 64 to 2 mg/L against 5,032 global clinical isolates, with a susceptibility rate of 98.3% at the susceptibility breakpoint of 4/4 mg/L (20). For all antimicrobial-non-susceptible phenotypes of *A. baumannii*, including carbapenem- and colistin-resistant isolates, the susceptibility rate of sulbactam-durlobactam was >96% (20). With respect to sulbactam-durlobactam non-susceptibility, a complete understanding of resistance mechanisms in *A. baumannii* remains to be elucidated, but two primary mechanisms have emerged from the sequencing of sulbactam-durlobactam-non-susceptible isolates. As durlobactam has poor inhibitory activity against the Class B metallo-beta-lactamases (MBL), expression of New Delhi MBL-1 (NDM-1) is associated with high-level resistance to sulbactam-durlobactam, with observed MIC values >32/4 mg/L (21–23). Though the prevalence of MBLs in *A. baumannii* isolates is exceedingly low in the United States, the threat of NDM-producing CRAB may be higher in Latin America and the Middle East (24). Mutations in PBP3, the key target site for sulbactam, represent a more commonly identified pattern of reduced activity. The most frequently reported PBP3 mutations impacting sulbactam-durlobactam activity are A515V, Q488K and Y258H (co-produced), and T526S substitutions, and while MIC values are elevated and often non-susceptible, these mechanisms are usually associated with lower MICs as compared to MBL-producers (25, 26). As many non-susceptible isolates have not undergone molecular testing in *in vitro* studies, the mechanisms conferring sulbactam-durlobactam resistance are still incompletely understood, and there is some evidence that mutations in the gene encoding the AdeJ efflux pump may also lead to reduced susceptibility (19, 26).

The clinical efficacy of sulbactam-durlobactam was evaluated in the phase III, multi-center ATTACK trial, which included patients hospitalized with pneumonia or bloodstream infections due to CRAB (27). Patients were randomized to receive either 1 g (both components) of sulbactam-durlobactam intravenously (IV) every 6 h infused over 3 h or 2.5 mg/kg colistin IV every 12 h for 7–14 days. All patients in the study received 1 g imipenem IV q6h in combination with their assigned study drug, given that the Gram-negative activity of sulbactam-durlobactam is largely limited to *A. baumannii,* combined with the need to cover for other potential Gram-negative pathogens empirically and the relative discomfort with polymyxin monotherapy. The primary efficacy outcome was assessed in the microbiologically modified intention-to-treat population, consisting of patients with eligible infection types that received a dose of study drug and whose *A. baumannii* isolates were confirmed carbapenem-resistant but retained susceptibility to both sulbactam-durlobactam and colistin. The primary outcome of 28-day all-cause mortality was assessed in 125 patients, and sulbactam-durlobactam demonstrated non-inferiority to colistin with a mortality rate of 19.0% compared to 32.3% (treatment difference −13.2%; 95% confidence interval [CI] −30.0% to 3.5%), respectively. Sulbactam-durlobactam was also associated with significantly higher rates of clinical cure at test of cure (62% vs 40%, 22% treatment difference, 95% CI 2.9% to 40.3%) and lower rates of RIFLE-defined acute kidney injury (13% vs 38%; *P* < 0.01) compared to colistin-treated patients.

While the relatively small sample size of ATTACK and the lack of additional clinical trial data may limit the level of confidence in the efficacy of sulbactam-durlobactam, it is worth noting that cefiderocol failed to demonstrate similar results versus best-available therapy (BAT, also largely colistin-based regimens) in the subgroup of patients with CRAB infections in the CREDIBLE-CR trial (28). In fact, 28-day mortality was 16/42 (38%) in the cefiderocol arm versus 3/17 (18%) in the BAT arm. Therefore, while the data supporting sulbactam-durlobactam are limited, they provide the strongest evidence to date of a clinical benefit for any antibacterial agent for invasive CRAB infections and support first-line use of sulbactam-durlobactam for such infections. Accordingly, IDSA guidance now recommends sulbactam-durlobactam as the preferred therapy for severe CRAB infections, with the caveat that therapy still be administered in combination with a carbapenem due to the conditions under which the clinical trials were conducted (10).

## IS COMBINATION THERAPY WITH A CARBAPENEM *REALLY* NECESSARY WHEN TREATING CRAB?

Despite having *in vitro* potency against CRAB, sulbactam-durlobactam has a relatively limited spectrum of antibacterial activity against other gram-negative pathogens. Likely owing to direct PBP2 inhibition, durlobactam itself displays intrinsic activity against *Escherichia coli*, *Klebsiella pneumoniae*, *Enterobacter cloacae*, *Citrobacter* spp., and *Stenotrophomonas maltophilia* to varying degrees, potentially expanding the antibacterial spectrum of sulbactam (18). Despite these promising *in vitro* results, sulbactam-durlobactam does not have activity against *Pseudomonas aeruginosa*, one of the most prevalent respiratory pathogens in nosocomial pneumonia. Given the absence of evidence supporting PBP2 inhibitors for invasive infections (discussed more below) and this lack of *P. aeruginosa* activity, sulbactam-durlobactam could not be utilized as a stand-alone agent in the ATTACK trial, particularly considering that the incidence of polymicrobial infections in recent clinical trials of patients with nosocomial pneumonia has ranged from 20% to 40% (27, 29–31).

While it therefore makes sense as to why combination therapy with imipenem was included in the clinical trial, the question facing clinicians is whether they must give a carbapenem in combination with sulbactam-durlobactam in clinical practice, as the IDSA guidance document recommends. Mechanistically, there is a basis for enhanced activity with the imipenem-sulbactam-durlobactam combination, given the complementary PBP binding of sulbactam (principally PBP3 and to a lesser degree PBP1b), and imipenem (principally PBP2 and to a lesser degree PBP1a) potentially leading to an enhanced effect when both compounds are protected from hydrolysis by durlobactam (32). From an MIC standpoint, the addition of imipenem does little to the activity of sulbactam-durlobactam against susceptible strains of CRAB. Assessing the 175 baseline CRAB isolates in the ATTACK trial, the MIC_50_ and MIC_90_ values of sulbactam-durlobactam (2/4 and 4/4 mg/L, respectively) and imipenem-sulbactam-durlobactam (1/4 and 4/4 mg/L, tested in a 1:1 ratio of imipenem: sulbactam) were similar (19). Conversely, when focusing on sulbactam-durlobactam non-susceptible strains in the trial (*n* = 8, all with PBP3 mutations, sulbactam-durlobactam MIC range of 8/4–16/4 mg/L), the addition of imipenem lowered the sulbactam-durlobactam MIC value below the CLSI breakpoint of 4/4 mg/L for seven of these eight isolates.

Taken together, these *in vitro* data support greater potency with the addition of a carbapenem to sulbactam-durlobactam in CRAB isolates with low-level resistance to sulbactam-durlobactam, possibly as a result of PBP2 saturation by the carbapenem becoming more important in the setting of PBP3 mutations impacting sulbactam binding. The data are less clear in sulbactam-durlobactam-susceptible isolates. Although MICs are only modestly reduced with the addition of imipenem in this setting, MIC testing might not be the best methodology to assess the impact of PBP-based synergy on activity. Indeed, a recent analysis by Veeraraghavan et al. demonstrated potent synergy and enhanced activity of the imipenem-sulbactam-durlobactam combination in time-kill studies against sulbactam-durlobactam-susceptible CRAB, even in the absence of significant MIC changes (32). Intriguingly, the authors demonstrated similar synergy with imipenem-sulbactam (without durlobactam, given the absence of carbapenemases) without lowering the MIC in a carbapenem-susceptible isolate, supporting the hypothesis that complementary PBP binding rather than imipenem serving as a substrate to tie up OXA carbapenemases is the mechanism for the enhanced effect. Notably, however, these time-kill studies were performed at sub-therapeutic concentrations, and thus, the clinical relevance of these interactions is unclear. Therefore, the available *in vitro* data are insufficient to determine if the addition of a carbapenem played a role in the outcomes demonstrated in ATTACK, and the impact of an additive carbapenem with respect to both clinical outcomes and resistance development, both in sulbactam-durlobactam-susceptible and PBP3-mutant CRAB isolates, is unknown and warrants further investigation.

In the absence of clinical data, a thorough assessment of the PK/PD relationship between sulbactam-durlobactam and CRAB can provide useful insights on the necessity of a carbapenem. That is, if the PK/PD data are robust and target exposures are routinely achieved with labeled dosing, clinicians can have an increasing degree of comfort with sulbactam-durlobactam monotherapy. A summary of the PK/PD analyses performed for durlobactam in combination with sulbactam can be found in Table 1. First, dose fractionation studies in a hollow fiber infection model performed on a CRAB isolate with sulbactam and sulbactam-durlobactam MIC values of 16 and 4/4 mg/L, respectively, identified 24 h area under the plasma concentration-time curve (AUC_24h_/MIC) as the optimal durlobactam PK/PD index (33). When assessed in the background of a sulbactam exposure reflecting an fT > MIC of 85%, the investigators demonstrated that durlobactam AUC_24h_/MIC values of 13.8 and 24.2 were associated with 1-log_10_ and 2-log_10_ CFU reduction, respectively. The same CRAB isolate was then used to inform the durlobactam PK/PD target in a one-compartment *in vitro* infection model. In this model, the investigators determined the durlobactam AUC_24h_/MIC target associated with activity in the backdrop of simulated exposures representing 2 g of sulbactam every 6 h. The investigators found similar AUC_24h_/MIC targets for durlobactam with an AUC_24h_/MIC of 7.6 and 33.4 to be necessary for 1-log_10_ and 2-log_10_ CFU reductions (33).

**TABLE 1:** Summary of *in vitro* and *in vivo* model PK/PD exposure targets for durlobactam against carbapenem-resistant *Acinetobacter baumannii^a^*

Model	No. isolates	SUL-DUR MIC range^*b*^ (mg/L)	Sulbactam exposure	R^2^	Durlobactam fAUC_24h_/MIC	
					1-log_10_ CFU reduction	2-log_10_ CFU reduction
*In vitro* HFIM (PVDF) (33)	1	4	85% fT > MIC	0.95	13.8	24.2
*In vitro* one-compartment chemostat (33)	1	4	2 g q6h	0.86	7.6	33.4
*In vivo* thigh (4:1 fixed ratio of SUL:DUR dosing) (34)	5	0.5–4	33%–44% fT > MIC	0.86	8.0	16.0
*In vivo* lung (4:1 fixed ratio of SUL:DUR dosing) (34)	4	0.5–4	44%–82% fT > MIC	0.91	10.6	22.4
*In vivo* thigh (fixed SUL dose^*c*^) (34)	6	0.5–16	18%–53% fT > MIC	0.82	7.5	31.8

^*a*^ AUC_24h_/MIC, 24-h area under the plasma concentration-time curve; CFU, colony-forming units; SUL, sulbactam; DUR, durlobactam; MIC, minimum inhibitory concentration; HFIM, Hollow Fiber Infection Model; PVDF, polyvinylidene fluoride.

^*b*^ Modal MIC of sulbactam in the presence of a constant 4 mg/L durlobactam.

^*c*^ In this model, the investigators used a fixed dose of sulbactam to achieve fT > MIC exposures ranging from 18% to 53% while varying the durlobactam exposures to identify the durlobactam PK/PD target.

While these two *in vitro* models are largely in agreement over the magnitude of durlobactam exposure necessary to restore 1-log_10_ kill with sulbactam (AUC_24h_/MIC of 7.6–13.8), these targets were derived in the setting of sulbactam exposures that are unlikely to be reflective of clinical practice. As the durlobactam PK/PD target necessary to restore the activity of sulbactam will be dependent on the amount of sulbactam present (i.e., the more sulbactam present, the less inhibitory activity is needed from durlobactam to achieve stasis and 1-log_10_ reduction endpoints), the use of higher sulbactam doses (2 g q6h instead of the labeled 1 g q6h dose) and potentially a higher fT > MIC exposure than can be achieved depending on the isolate MIC (fT > MIC of 85%) limits the translatability of these data. Additionally, the use of a single CRAB isolate to inform both models may also fail to demonstrate variability in the PK/PD relationship that could exist among a broader range of susceptibilities. As such, the clinical interpretability of findings from these *in vitro* models to determine an optimal durlobactam dosing target is unclear.

The PK/PD was further explored using plasma exposures in murine neutropenic thigh and lung infection models in order to identify targets for both sulbactam when used in combination with durlobactam and durlobactam when used in combination with sulbactam against CRAB (34). Co-modeling of sulbactam alone against a single sulbactam-susceptible isolate and at a fixed 4:1 dose of sulbactam-durlobactam against multiple CRAB isolates demonstrated that a sulbactam fT > MIC of 29.9% and 38.2% in the thigh model, and 41.2% and 53.5% in the lung model were associated with a 1-log_10_ and 2-log_10_ kill, respectively. In the 4:1 dose titration model evaluating durlobactam exposure magnitudes to restore sulbactam activity against CRAB isolates, a fAUC_24h_/MIC of 8.0 and 16.0 in the thigh model and 10.6 and 22.4 in the lung model restored 1-log_10_ and 2-log_10_ kill, respectively, which was largely consistent with the *in vitro* modeling (Table 1). A fixed 4:1 titration was specifically chosen with the intention of matching exposures necessary for antibacterial activity from the *in vitro* PK/PD model; however, the clinical applicability of this approach may be limited as these drugs are administered in a 1:1, not 4:1 ratio. Therefore, while all evaluated models placed the durlobactam fAUC_24h_/MIC target in the vicinity of 10 to restore 1-log_10_ reductions with sulbactam, the clinical applicability of this estimate remained questionable.

However, an additional analysis that was performed in the neutropenic thigh model provides more clinically relevant insight regarding the durlobactam exposures required to restore the activity of sulbactam. In this model, the investigators used a fixed dose of sulbactam to achieve fT > MIC exposures ranging from 18% to 53% against six strains ranging in MICs to sulbactam-durlobactam from 0.5/4 to 16/4, which are both clinically relevant exposures and consistent with the fT > MIC target of 50% that was utilized for joint probability of target attainment assessment (described below) (34). Using this methodology, the authors demonstrated that similar durlobactam fAUC_24h_/MIC values of 7.5 and 31.8 were the identified targets to restore 1-log_10_ kill and 2-log_10_ kill with sulbactam, respectively. Importantly, the magnitude of these targets was nearly identical across the range of sulbactam-durlobactam MIC values tested, reflecting a proportional relationship between exposure and susceptibility.

With these preclinical PK/PD findings, a 50% fT > MIC for sulbactam and an fAUC_24h_/MIC of 10 for durlobactam were selected as the PD targets against CRAB and thus applied in PTA analyses (35). When considering differential ELF penetration between mice and humans (34, 36), and population PK analyses from eight Phase I–III clinical trials (37, 38), Monte Carlo simulations were performed to assess the joint probability of target attainment for sulbactam and durlobactam using the package insert dosing across renal function strata. Although these data are not published in manuscript form, an abstract of these findings indicates that ≥90% PTA is achieved for all renally adjusted dosing regimens for sulbactam-durlobactam MIC values up to the susceptibility breakpoint of 4/4 mg/L in both plasma and ELF (37). At an MIC of 8/4 mg/L, PTA drops considerably below 90% in patients with normal renal function, suggesting that sulbactam-durlobactam monotherapy should not be relied upon in isolates with potential PBP3 variants demonstrating low-level non-susceptibility. In fact, an assessment of ELF exposures in the HFIM demonstrated that 1 g sulbactam q6h (32% fT > MIC) and 1 g durlobactam q6h (estimated fAUC_24h_/MIC of 19.5) did not achieve 24 h bacterial stasis in two PBP3-mutant CRAB isolates with sulbactam-durlobactam MIC values of 8/4 mg/L (33). Importantly, the addition of 1 g meropenem q6h or 1 g imipenem q6h to both isolates resulted in a log_10_-CFU reduction similar to what was observed for sulbactam-durlobactam alone against a CRAB isolate with a sulbactam-durlobactam MIC of 0.5 mg/L, on the order of 3- to 5-log_10_ CFU reduction from baseline.

Taken together, these PK/PD data suggest that sulbactam-durlobactam should be effective for the treatment of CRAB with MIC values up to 4/4 mg/L. Furthermore, the addition of a carbapenem may be most important when isolates test intermediate at a sulbactam-durlobactam MIC of 8/4 mg/L. However, no comparative efficacy data exist assessing sulbactam-durlobactam alone and in combination with a carbapenem for the treatment of CRAB. While the PK/PD supports omitting the carbapenem for susceptible isolates, available *in vitro* data support the additive benefit of a carbapenem on the activity of sulbactam-durlobactam, and the limited clinical data only assess sulbactam-durlobactam in combination with imipenem. Given this, while it remains possible, and perhaps even likely, that the efficacy demonstrated in the ATTACK trial was driven by sulbactam-durlobactam alone, it is also possible that the effect of sulbactam-durlobactam—even if efficacious on its own—was enhanced by the addition of the carbapenem. Therefore, until the two strategies are compared clinically, our preference is to combine sulbactam-durlobactam with a carbapenem for invasive infections and critically ill patients. However, sulbactam-durlobactam monotherapy may be considered for patients with less severe infections due to susceptible CRAB monomicrobial infections, such as wound or urinary tract infections, in order to limit potentially unnecessary carbapenem usage.

## IS THERE A ROLE FOR SULBACTAM-DURLOBACTAM BEYOND *A. BAUMANNII*?

As previously mentioned, durlobactam exhibits intrinsic antibacterial activity against *E. coli* and other Enterobacterales via direct PBP2 inhibition. One clinical scenario where this may be important is in the treatment of the increasingly prevalent global threat of NDM-producing *E. coli*. When tested against 32 carbapenemase-producing Enterobacterales isolates, including MBL-producing strains, MIC values for durlobactam ranged from 0.125 to 8 mg/L (18). Current guidance supports the use of cefiderocol or a combination of aztreonam and ceftazidime-avibactam to target isolates displaying this resistance genotype, with both therapies exerting activity principally against PBP3. Despite having stability against NDM-mediated hydrolysis, PBP3 insertions frequently identified in NDM-producing *E. coli* hinder the binding affinity of both aztreonam and cefiderocol, resulting in reduced susceptibility (39, 40). A now growing body of evidence demonstrates that NDM-producing *E. coli* isolates have concerningly high rates of cefiderocol and aztreonam-avibactam non-susceptibility, with susceptibility rates reported to be as low as 34% and 80%, respectively (41). PBP3 insert mutations only partially explain the reduced activity of aztreonam-avibactam, with co-harbored CMY variants further leading to an inability of avibactam to fully protect aztreonam from hydrolysis (39). Though durlobactam is not capable of inhibiting NDM enzymes, it is stable against hydrolysis, and its PBP2-mediated *in vitro* activity makes it an intriguing complementary agent in isolates with reduced susceptibility to cefiderocol or aztreonam-avibactam, for mechanistically similar reasons to the aforementioned benefit of adding a carbapenem to sulbactam-durlobactam for CRAB isolates with elevated sulbactam-durlobactam MICs due to PBP3 mutations. Durlobactam has been shown to consistently retain low MIC values ranging from 0.125 to 2 mg/L against NDM-producing *E. coli*, with respective MIC_50_ and MIC_90_ values of 0.25 mg/L and 1 mg/L (41, 42).

Importantly, there are potential concerns surrounding the clinical use of durlobactam, considering the high frequency of resistance development observed for other PBP2 inhibitors such as mecillinam (43). However, use in combination with agents such as cefiderocol or aztreonam-avibactam and their complementary PBP3 binding activities may mitigate resistance development and preserve activity. Indeed, cefepime-zidebactam is an analogous agent in development that has demonstrated potent *in vitro* and *in vivo* activity against isolates producing NDM with PBP3 inserts, despite cefepime’s lability to hydrolysis by NDM and decreased binding in the presence of PBP3 mutations, presumably due to zidebactam’s ability to inhibit PBP2 (44). Although clinical data for durlobactam are lacking against NDM-producing *E. coli*, a secondary analysis of the ATTACK trial assessing the efficacy of sulbactam-durlobactam in combination with imipenem in 12 patients with polymicrobial infections with CRAB and imipenem-resistant (MIC ≥ 4 mg/L) *K. pneumoniae* is intriguing (45). Durlobactam displayed notable *in vitro* activity (MIC range 1–8 mg/L) against 11/12 isolates and reduced the imipenem MIC to 0.5–2 mg/L in 10/12 isolates. Twenty-eight-day mortality in this subset of patients was 2/12 (17%), and clinical cure was 9/12 (75%), with both rates being consistent with outcomes in patients with monomicrobial CRAB infections. Whether efficacy in these patients was driven by direct antibacterial activity of durlobactam, beta-lactamase inhibition restoring the activity of imipenem, a combination of both mechanisms, or was merely an incidental finding given the small sample size is unclear. Therefore, while it is premature to recommend durlobactam in such scenarios, further evaluation of *in vitro* activity, PK/PD, and *in vivo* efficacy against Enterobacterales is warranted.

With respect to *P. aeruginosa*, while durlobactam can produce morphological changes that may alter its virulence, it does not demonstrate independent antibacterial activity *in vitro* as is seen with Enterobacterales (18). On the other hand, durlobactam may have a particular niche over other DBOs as a BLI in some MDR *P. aeruginosa* infections. Against isogenic strains of *P. aeruginosa* producing OXY-6-2 and PER-4, durlobactam potentiated cefepime activity *in vitro* to a greater extent than vaborbactam, avibactam, and relebactam, and as expected with its spectrum of inhibition, durlobactam also improved activity of meropenem and cefepime in strains producing Class A, C, and D beta-lactamases and carbapenemases (46). In the previously described secondary analysis of the ATTACK trial, the investigators assessed outcomes in three patients treated with sulbactam-durlobactam in combination with imipenem for a polymicrobial infection caused by CRAB and imipenem-resistant *P. aeruginosa* (all three patients’ Pseudomonas isolates had imipenem MIC values of 16 mg/L) (45). The addition of durlobactam (4 mg/L) restored *in vitro* susceptibility in all three isolates (MIC 1–2 mg/L). This reduction in imipenem MICs is similar to what is demonstrated when relebactam is combined with imipenem against imipenem-resistant *P. aeruginosa*. All three patients survived, with two demonstrating clinical cure (the third had an indeterminate outcome). Perhaps even more interesting are the recent data from Dorazio et al. assessing the ability of currently available DBOs (avibactam, relebactam, and durlobactam) to restore activity of ceftolozane-resistant Difficult to Treat Resistant (DTR) *P. aeruginosa* caused by mutations potentiating ampC-mediated hydrolysis of ceftolozane. In an assessment of pre- and post-treatment clinical *P. aeruginosa* isolates that developed resistance while on ceftolozane-tazobactam, ceftolozane-durlobactam had the lowest MIC fold-changes from baseline when compared with the other DBOs, avibactam and relebactam, consistent with more potent activity of durlobactam against certain PDC variants (47). While enhancing the activity of various beta-lactams against *P. aeruginosa* with durlobactam is not a strategy that is ready for prime time, clinicians should be aware of the potential benefits for one-off clinical scenarios, and further evaluation of durlobactam in this setting is warranted.

## CONCLUSION

Safe and effective treatment options for severe infections due to CRAB were greatly limited prior to the approval of sulbactam-durlobactam, which has immediately been adopted as the drug-of-choice in this clinical space. *In vitro* data demonstrate potent activity of sulbactam-durlobactam against CRAB, with high-level resistance moderated by MBL expression currently being uncommon. Though comparative clinical data are relatively limited to date, sulbactam-durlobactam has shown more promising results than cefiderocol for CRAB in similarly sized randomized clinical trials. The CRAB PD targets of 50% fT > MIC for sulbactam and fAUC_24h_/MIC of 10 for durlobactam are supported with agreement between *in vitro* and *in vivo* PK/PD analyses, and preliminary MCS suggests appropriate PTA of labeled dosing up to the susceptibility breakpoint of 4/4 mg/L. While *in vitro* data clearly support a role for carbapenem combination therapy when the MIC demonstrates low-level non-susceptibility to sulbactam-durlobactam, the role of combination therapy with a carbapenem in susceptible isolates is less clear. Until further clinical data become available, we agree with the IDSA recommendation for carbapenem combination therapy in patients with invasive CRAB infections. Durlobactam, as a PBP2 inhibitor in Enterobacterales and a DBO BLI with potent Class A, C, and D enzyme inhibition, may also serve niche therapeutic roles as last-line therapy against NDM-producing *E. coli* with PBP3 insert mutations or as combination therapy to restore beta-lactam activity against DTR *P. aeruginosa*. However, further evidence is clearly needed before either of these strategies can be recommended.
